# Prognosis and Therapy When Acute Promyelocytic Leukemia and Other “Good Risk” Acute Myeloid Leukemias Occur as a Therapy-Related Myeloid Neoplasm

**DOI:** 10.4084/MJHID.2011.032

**Published:** 2011-07-08

**Authors:** Richard A. Larson, Michelle M. Le Beau

**Affiliations:** Department of Medicine and Comprehensive Cancer Center, University of Chicago, Chicago IL, USA

## Abstract

Treatment for a pre-existing condition using chemotherapy, radiation therapy, immunosuppressive therapy, or a combination of these modalities may lead to the devastating complication of therapy-related myelodysplastic syndrome or acute myeloid leukemia (t-MDS/t-AML), collectively known as therapy-related myeloid neoplasm (t-MN). This disorder arises as a direct consequence of mutational events induced by the primary treatment. The outcomes for these patients have been historically poor compared to people who develop AML *de novo*. Currently comprising 10–20% of all cases of AML, t-MN is relatively resistant to conventional leukemia therapies, and is associated with s ort survival times. Median life expectancy from diagnosis is about 8–10 months in most series. Although the spectrum of cytogenetic abnormalities in t-AML is similar to AML *de novo*, the frequency of unfavorable cytogenetics, such as a complex karyotype or deletion or loss of chromosomes 5 and/or 7, is considerably higher in t-MN. Two distinct groups of patients with t-MN have been described. The more common subtype, seen in about 75% of patients, typically occurs 5–7 years after first exposure to alkylating agents or radiation, is often preceded by a myelodysplastic syndrome (MDS), and is frequently accompanied by clonal cytogenetic abnormalities such as the loss of all or part of chromosomes 5 or 7. Mutations of the *P53* tumor suppressor gene are also common. The risk is related to total cumulative exposure over time to alkylating agents. In contrast, among individuals who develop t-AML after treatment with topoisomerase II inhibitors, the latency period to the development of t-AML is often only 1–3 years, antecedent MDS is rare, and gene rearrangements involving *MLL* at 11q23 or *RUNX1/AML1* at 21q22 are common. It is now well recognized that APL and other subtypes of AML with balanced translocations sometimes occur as therapy-related myeloid neoplasms (t-MN) in patients who have previously received cytotoxic therapy or ionizing radiation therapy (RT). The most of this review will focus on these “good risk” leukemias, i.e. those with APL or inv(16)/t(16;16) or t(8;21).

***Case Presentation:****A 46-year-old woman was diagnosed with localized breast cancer. She underwent primary excision followed by adjuvant chemotherapy with doxorubicin and cyclophosphamide followed by paclitaxel; filgrastim was given to maintain a dose-dense schedule. Finally, she received radiation therapy to the left chest wall. No recurrence of breast cancer has been detected on routine follow up and imaging. However, 3 years later, she was noted to have pancytopenia. A bone marrow examination revealed a markedly hypercellular marrow with 90% malignant promyelocytes and blasts. The karyotype was 46XX,t(15;17) in all 30 metaphase cells. Her cardiac left ventricular ejection fraction has declined to 40%.*

Is this therapy-related acute promyelocytic leukemia (t-APL)? Will it respond differently than *de novo* APL? Can it be treated successfully without exposure to additional anthracyclines or the use of hematopoietic stem cell transplantation (HCT)? Do “cytogenetically favorable” subsets of therapy-related myeloid leukemia (t-AML) have a different outcome than more typical cases of t-AML with complex cytogenetic abnormalities?

## The Syndrome of Therapy-Related Myeloid Neoplasm:

Treatment for a pre-existing condition using chemotherapy, radiation therapy, immunosuppressive therapy, or a combination of these modalities may lead to the devastating complication of therapy-related myelodysplastic syndrome or acute myeloid leukemia (t-MDS/t-AML), collectively known as therapy-related myeloid neoplasm (t-MN).^1^ This disorder arises as a direct consequence of mutational events induced by the primary treatment. The outcomes for these patients have been historically poor compared to people who develop AML *de novo*. Currently comprising 10–20% of all cases of AML, t-MN is relatively resistant to conventional leukemia therapies, and is associated with short survival times.^1^–^3^ Median life expectancy from diagnosis is about 8–10 months in most series.^2^ Although the spectrum of cytogenetic abnormalities in t-AML is similar to AML *de novo*, the frequency of unfavorable cytogenetics, such as a complex karyotype or deletion or loss of chromosome 5 and/or 7, is considerably higher in t-MN.^2^

Two distinct groups of patients with t-MN have been described.^1^,^2^,^4^,^5^ The more common subtype, seen in about 75% of patients, typically occurs 5–7 years after first exposure to alkylating agents or radiation, is often preceded by a myelodysplastic syndrome (MDS), and is frequently accompanied by clonal cytogenetic abnormalities such as the loss of all or part of chromosomes 5 or 7. Mutations of the *P53* tumor suppressor gene are also common. The risk is related to total cumulative exposure over time to alkylating agents. In contrast, among individuals who develop t-AML after treatment with topoisomerase II inhibitors, the latency period to the development of t-AML is often only 1–3 years, antecedent MDS is rare, and gene rearrangements involving *MLL* at 11q23 or *RUNX1/AML1* at 21q22 are common. Risk is less clearly related to total cumulative dose but is associated with chemotherapy dose and schedule.

## The Importance of Cytogenetic Abnormalities in Predicting Patient Outcomes:

Within a series of 306 patients with t-MN studied at the University of Chicago, the median survival rate was approximately 7 to 9 months, and varied with karyotype.^2^ The longest median overall survival rates were seen in patients with normal karyotypes or recurring balanced rearrangements (approximately 11 months each). However, the incidence of unfavorable karyotypes was greater than 70%. The shortest median survival was seen in patients with abnormalities of both chromosomes 5 and 7 (approximately 5 months). Only 24 patients (8%) were alive 3 years after diagnosis. Patients with t-MN who responded to remission induction therapy but subsequently died from their primary malignancy were included in the survival analysis. The short survivals reported in this early series compared with more recent series described below reflects a higher percentage of patients who received only supportive care rather than undergo remission induction chemotherapy at that time.

Survival for patients receiving intensive remission induction chemotherapy varies according to cytogenetic risk group. Better outcomes are observed in t-MN patients with more favorable-risk karyotypes. A large comparative analysis reported by the German AML Cooperative Group included 93 patients with t-AML and 1091 patients with *de novo* AML treated with standard AML induction therapy.^6^ Overall, the median survival was 10 months for patients with t-AML compared to 15 months for patients with *de novo* AML (P=0.0007). Favorable, intermediate, and unfavorable karyotypes were observed in 26%, 28%, and 46% of t-AML patients, and in 22%, 57%, and 20% of *de novo* AML patients. The high frequency of adverse cytogenetics may explain to a large extent the unfavorable outcomes of patients with t-AML. Although favorable and unfavorable cytogenetics had prognostic value in both patient groups, the survival of patients with t-AML was generally shorter than that of those with *de novo* AML within the same cytogenetic risk group. When updated to include 121 patients with t-AML, the median overall survival times for patients with t-AML with favorable, intermediate, and unfavorable cytogenetics were 27, 13, and 6 months, respectively (Table 1).^7^ For those with a favorable karyotype, the median survival was not yet reached after 5 years for the 306 *de novo* AML patients compared to 27 months for the 29 t-AML patients (P=0.02). Some of these t-AML patients appeared to be cured. Within the large intermediate risk cytogenetic group, no significant difference in survival was observed between the t-AML and *de novo* AML patients. An unfavorable karyotype predicated a very short survival in both groups of AML patients.

Armand et al analyzed the outcomes of 80 patients with therapy-related leukemia treated at the Dana Farber Cancer Institute in Boston.^8^ They found that cytogenetic abnormalities were the strongest predictors for overall survival. When adjusted for cytogenetic changes, the patients with t-AML did as well as patients with *de novo* AML, further emphasizing the importance of cytogenetic abnormalities in predicting severity of disease and outcomes.

Kayser et al recently reported on 200 patients with t-AML and compared their clinical and biological characteristics and outcomes with those of 2653 patients with *de novo* AML treated on 6 prospective multi-center trials by the German-Austrian AML Study Group.^3^ Seventy-five percent of the t-AML cases had an abnormal karyotype compared to 51% of the *de novo* cases, and 15% of the t-AML cases had either a t(15;17) [2%], t(8;21) [5%], or inv(16) or t(16;16) [8%]. The t-AML patients were older (median, 58 vs 53 years; p<0.0001) and more often female (68% vs 47%; p<0.0001). The median latency was 4.0 years (range, 0.3 to 44 years). The response to remission induction therapy was 63% CR for t-AML and 67% for *de novo* AML (p=0.21). However, the outcome for t-AML patients was significantly inferior. Relapse-free survival at 4 years was 25% for t-AML and 40% for *de novo* AML (p<0.0001). In multivariable models adjusted for white blood cell counts, among patients with an inv(16) or t(16;16), t-AML was a significant adverse prognostic factor for overall survival (hazard ratio 2.35; p=0.04).

## Factors That Influence Outcome in t-MN:

Therapy-related myeloid leukemia is generally a fatal disease. The life-threatening complications of this disorder are the result of persistent and profound cytopenias due to the failure of normal hematopoiesis regardless of the fraction of myeloblasts accumulating in the bone marrow or blood. There has been general agreement that patients with t-AML have shorter survivals than patients with de novo AML. Supportive care is still considered by many to be the standard management.

The survival of patients with t-MN is often poor despite prompt diagnosis and treatment. However, there is a paucity of prospective treatment data since these patients are most often excluded from frontline clinical trials. There are no randomized studies comparing standard AML therapy to other forms of treatment. A number of potential factors explain the poor outcome of patients with t-MN. Poor hematopoietic reserves can make the administration of standard AML therapy difficult. Because t-MN evolves in the milieu of chemotherapy, the malignant cells may be more drug-resistant than in *de novo* disease. Persistence of the primary malignant disease, particularly metastatic cancer or lymphoma, causes morbidity and mortality independent of the bone marrow failure caused by leukemia. Many patients have poor tolerance for the acute toxicity of treatment. Injury to organs and their vascular supply from prior treatment may compromise the ability of these patients to receive intensive chemotherapy or successful HCT. There may be depletion of normal hematopoietic stem cells as a consequence of previous therapy, so that these patients suffer prolonged cytopenias after induction chemotherapy. The bone marrow stroma may have been damaged, especially by therapeutic radiation to fields that include the pelvis or lumbosacral spine, so that it will not support regeneration of normal hematopoiesis. Patients with t-AML are often chronically immunosuppressed from prior disease or on-going therapy or may have dysfunctional and dysplastic phagocytes, and thus are often colonized with pathogenic or antibiotic-resistant bacteria and fungi. Following prior supportive care, patients may be refractory to additional transfusion support, and therefore, not good candidates for intensive myelosuppressive chemotherapy. Finally, the high frequency of unfavorable cytogenetic aberrations arising during or after chemoradiotherapy appears to result in the rapid emergence of chemotherapy resistance in t-AML stem cells. Relapses even after myeloablative chemoradiotherapy and allogeneic HCT are not uncommon.

## Outcomes of t-MN Patients with “Good Risk” Cytogenetics:

It is now well recognized that APL and other subtypes of AML with balanced translocations sometimes occur as therapy-related myeloid neoplasms (t-MN) in patients who have previously received cytotoxic therapy or ionizing radiation therapy (RT).^1^,^3^,^9^ The exact mechanism of the leukemogenic transformation remains to be determined. The remainder of this review will focus on “good risk” leukemias, i.e. those with APL or inv(16)/t(16;16) or t(8;21).

Among 511 patients analyzed at the International Workshop on the Relationship of Prior Therapy to Balanced Chromosome Aberrations in Therapy-Related Myelodysplastic Syndromes and Acute Leukemia held in Chicago in 2000, chromosome 21q22 rearrangements were seen in 79 (∼15%), inv(16) in 48 [9%; 2 patients had t(16;16)], and t(15;17) in 41 (8%).^10^ It was noted that 30% of t(15;17) cases and 21% of inv(16) cases had received only RT as their primary treatment, and this was significantly more frequent than in the chromosome 11q23 (5%) and 21q22 (7%) cases (p<0.001). The majority of patients in all subgroups had received alkylating agents. The chromosome 11q23 and 21q22 subgroups actually had the highest exposures to alkylating agents (86% and 82%, respectively); this was significantly greater than for the inv(16) and t(15;17) subgroups (63% and 59%, respectively). None of the t-APL patients had received the dioxypiperazine derivative, bimolane, once used in a Chinese study for treatment of psoriasis, and associated with t(15;17).

The disease was overt t-AML in 38 of 48 patients (79%) with an inv(16) and in 38 of 41 patients (93%) with a t(15;17).^11^ Cases with inv(16) had a short latency period (median, 22 months) and a long median survival, with 45% surviving for 5 years. The median latency for t(15;17) was 29 months, and the median survival 29 months. The primary disease for inv(16) cases was breast cancer (n=15), lymphoma(12),and other solid tumors (16). For the t(15;17) cases, it was breast cancer (18),lymphoma (11),and other cancers (12). Twenty-six patients with inv(16) (54%) and 17 with t(15;17) (41%) had additional cytogenetic abnormalities; these were unrelated to age or survival in both subgroups. Trisomy 8 was the most common additional abnormality in both; +21, +22 and +13 were common in the inv(16) subgroup, and −7/del(7q) and −5/del(5q) were present in a few cases in both subgroups.

## Outcomes of Patients with t-MN and T(15;17) or Inv(16):

Thirty-three of 39 patients (85%) with an inv(16) obtained a complete remission (CR) after intensive chemotherapy, but 12 of these subsequently relapsed.^10^ Four underwent HCT using bone marrow stem cells, and one underwent peripheral blood stem cell transplantation. All 5 of these patients remained in CR at the time of last follow-up. The 6 patients with inv(16) who did not respond to intensive chemotherapy were significantly older (median age, 62 years) as compared to the 33 responding patients (median, 44 years) (P=0.012).

Twenty-four of 35 patients (68%) with a t(15;17) obtained a CR after intensive chemotherapy, and 6 patients subsequently relapsed. Two additional patients achieved a CR, but follow up data were missing. Two patients received an allogeneic HCT, and one was in CR at last follow-up. In the t(15;17) subgroup, the 11 non-responding patients were similar in age to the 24 responding patients (medians, 48 and 50 years, respectively; p=0.99). Several patients with t(15;17) were treated with all-trans-retinoic acid (ATRA, tretinoin), but information on the use of ATRA was recorded inconsistently in the Workshop database.

The median survival time was 29 months in both cytogenetic subgroups, and many patients became long-term survivors. There were no significant differences in survival among intensively treated patients with or without additional chromosome abnormalities (log-rank test, p=0.16 for the inv(16) subgroup, and p=0.30 for the t(15;17) subgroup). The overall survival of 39 inv(16) patients with follow up data who had previously received chemotherapy with or without RT (n=33) for their primary disease was significantly better compared to patients who had received RT only (n=6) (log-rank test, P=0.03). The difference in survival between primary chemotherapy with or without RT and RT only was not statistically significant in the t(15;17) subgroup (P=0.45).

In the inv(16) subgroup, patients less than 55 years old had improved survival when compared to older patients. The median survival for the 26 younger patients had not been reached, but was only 12 months for the 13 older patients (log-rank, p=0.006). A similar tendency, although not statistically significant, was observed in the t(15;17) subgroup, with median survival times of 29 and 20 months in the younger (n=21) and older (n=15) cohorts (p=0.73), respectively.

## Treatment of Patients with t-MN and t(15;17) or inv(16):

In 2003, Beaumont and colleagues reported on 106 patients diagnosed with t-APL during the previous 20 years in 3 European countries; 80 were diagnosed within the previous 10 years.^12^ Primary disorders were predominantly breast cancer (n=60), non-Hodgkin lymphoma (15), and other solid tumors (25). Thirty patients had received chemotherapy alone, 27 had received RT alone, and 49 had received both. Prior chemotherapy included at least one alkylating agent in 68 patients and at least one topoisomerase-II inhibitor in 61, including anthracyclines (30), mitoxantrone (28),and epipodophyllotoxins (19).Median latency was 25 months (range, 4 to 276 months). Characteristics of t-APL were generally similar to those of *de novo* APL. Survival was 59% at 8 years.

Several additional series of patients with t-APL have recently been published. Jantunen and colleagues in Finland reported on 5 cases; all had received prior RT; 4 had received topoisomerase-II inhibitors with multiagent chemotherapy.^13^ All achieved CR with ATRA plus chemotherapy. One died from metastatic breast cancer and one relapsed; 3 remain in continuous CR after a median of 28 months. Dayyani and co-investigators at the MD Anderson Cancer Center reported on 29 patients with t-APL; 33% had received only prior RT.^14^ Their median age was about 54 years compared with 42 years for a cohort of *de novo* APL patients treated at the same institution (p<0.001).

Otherwise, clinical features and outcomes were similar. The CR rate was 89% for the 19 with t-APL who received ATRA plus arsenic trioxide (ATO), and 70% for 10 who received ATRA plus chemotherapy (p=0.35). At last follow up, 15 patients (52%) were alive; 9 had died from leukemia. Malhotra and colleagues in India treated 3 patients with t-APL with ATRA plus ATO for induction and consolidation.^15^ All 3 remain in molecular remission at 19–42 months.

In contrast to other subtypes of t-MN, patients who develop t-APL with t(15;17) or those with inv(16) have treatment outcomes that are similar to AML that arises *de novo* with the same chromosomal rearrangements. The combination of ATRA plus arsenic trioxide is an effective treatment for t-APL and may be particularly useful for patients who have diminished cardiac function from prior anthracycline exposure or radiation. Although non-leukemia co-morbidities or persistent primary malignancy still impact on ultimate survival, their leukemia should be treated similar to *de novo* disease.^16^

## Patients with t-MN and t(21q22):

The Workshop identified 79 t-MN patients with balanced chromosome 21q22 translocations; 44 (56%) had t(8;21), 11 as a sole abnormality and 33 in combination with other abnormalities.^17^,^18^ Median latency was 39 months overall. Patients had been treated for solid tumors (56%), hematologic malignancy (43%), and juvenile rheumatoid arthritis (1 case) with RT alone (n=5), chemotherapy alone (36), or combined-modality therapy (38). All 5 who had received only RT had t(8;21). Exposure to alkylating agents was significantly greater than for the inv(16) and t(15;17) subgroups (p<0.02 for both). Exposure to topoisomerase-II inhibitors was significantly greater than in the t(15;17) subgroup but similar to inv(16). Overt t-AML was present at diagnosis in 82% with t(8;21). Median survivals were 17 months for the 11 with only t(8;21) and 31 months for the 33 with t(8;21) plus other abnormalities (p=0.6). Mutations in *c-KIT* were not studied in these patients.^19^ Patients with t(8;21) had a more favorable outcome than those with other 21q22 present at diagnosis in 82% with t(8;21). Median survivals were 17 months for the 11 with only t(8;21) and 31 months for the 33 with t(8;21) plus other abnormalities (p=0.6). Mutations in *c-KIT* were not studied in these patients.^19^ Patients with t(8;21) had a more favorable outcome than those with other 21q22 rearrangements (p=0.014). Overall, median survival for the 21q22 patients was 14 months, and for patients with t(8;21) was 19 months.

**Figure 1:**
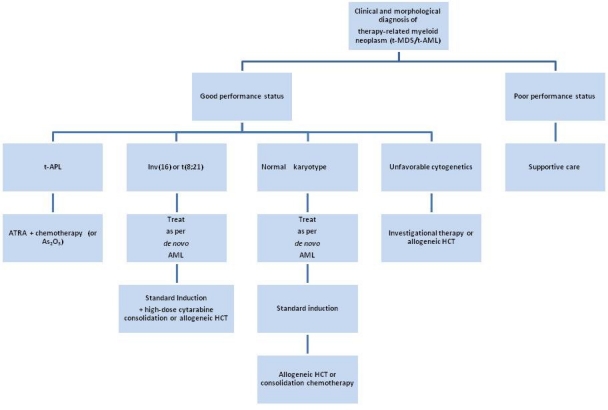


**Recommendations for Treatment of t-MN:** Figure 1 shows a treatment algorithm for the management of patients who develop therapy-related myeloid neoplasms. Primary considerations are the patient’s performance status, which reflects age, co-morbidities, the status of the primary disease, and the presence of complications from primary therapy, as well as the clonal abnormalities detected in the t-MN cells. Mutations in *c-KIT* were not studied in these patients.

In general, t-MN patients should be encouraged to participate in prospective clinical trials that are appropriately designed for other AML patients with similar cytogenetic abnormalities. Patients who have an HLA-matched donor should be considered for allogeneic HCT, although patients with favorable karyotypes such as t(15;17) and inv(16) may do well with conventional intensive chemotherapy.

## Figures and Tables

**Table 1.** Survival according to cytogenetic risk group for patients with t-AML or *de novo* AML treated by the German AML Cooperative Group (AMLCG)^7^

**Karyotype**	**No. of patients (%)**		**Median survival (months)**		
	t-AML (n=121)	*de novo* AML (n=1511)	t -AML	*de novo* AML	p
Favorable	29 (24)	306 (20)	27	>60	0.02
Intermediate	34 (28)	903 (60)	12	16	0.19
Unfavorable	58 (48)	302 (20)	6	7	0.006
